# The EAT-Lancet Commission's Dietary Composition May Not Prevent Noncommunicable Disease Mortality

**DOI:** 10.1093/jn/nxaa020

**Published:** 2020-02-15

**Authors:** Francisco J Zagmutt, Jane G Pouzou, Solenne Costard

**Affiliations:** EpiX Analytics, Fort Collins, USA

**Keywords:** planetary health diet, EAT-Lancet Commission, dietary recommendation, Monte Carlo simulation, stochastic modeling

## Abstract

The recently published EAT-Lancet Commission report on dietary impacts on the environment suggested that their proposed diet could prevent more than 10 million annual premature mortalities from noncommunicable diseases globally. The report did not meet standards for transparency and replicability, nor did it fully account for statistical uncertainty. Our attempt to replicate the mortality calculations for the United States revealed flaws in the assumptions and methods used to estimate the avoided mortalities. After correcting some calculation errors and fully accounting for uncertainty in the avoided mortalities, the mortality reduction effect of the EAT-Lancet proposed diet in the USA is no greater than the impact of energy consumption changes that would prevent under-weight, over-weight, and obesity alone. As our findings call into question the global conclusions of the EAT-Lancet report, futher independent validation is needed before it can be used to inform dietary guidelines.

On January 16, 2019 the EAT-Lancet Commission on healthy diets from sustainable food systems published its recommendation for a “universal healthy reference diet” (REF) (1). Although the sustainability of the proposed REF diet was evaluated, the daily dietary recommendations were based only on health considerations – measured through prevented mortality – and nutritional content, using methodology further described in Springmann et al. (2). The goal of reducing the global burden of noncommunicable diseases (NCD) associated with unhealthy diets (3) while working towards sustainable food systems is laudable; however, the approach presented in the EAT-Lancet report (referred to hereafter as the “report”) has several limitations that call the conclusions into question and caution against its use as standalone evidence for global nutritional policy recommendations.

In some sections, the report's language suggests that the REF diet is optimized for human health; the concept of optimal quantities of specific food groups is discussed, and the ranges of recommendations are described as “compatible with optimal health.” Yet, a formal mathematical optimization approach would be required to reach such conclusions, exploring a multitude of alternative diets with the objective of maximizing prevented mortality while respecting certain “constraints” (such as calorie intake, minimum and/or maximum daily amounts for some foods, or micronutrient composition). To construct a diet and then compare it against the constraints evaluates only one possible diet scenario and thus it may result in a suboptimal dietary solution, particularly for segments of the population with different dietary requirements or limited access to some dietary components. The use of optimization language suggests a more quantitative basis for the diet than appears to exist.

Also, the dietary risk factors driving the NCDs included in the report are most significant for middle, high-middle, and high sociodemographic index (SDI) countries, but in low-middle/low SDI countries, maternal and child malnutrition contribute 3.5 times more total disability-adjusted life years than all dietary factors combined (3). The result is a “global” diet that promotes social and nutritional inequity as it decreases mortality in the highest SDI countries the most, but only superficially addresses the issues of maternal and child health and food insecurity in areas such as Sub-Saharan Africa. The report only suggested that the role of animal food products be “carefully considered” in low-middle/low SDI countries, despite evidence of the benefits of animal products and integrated crop-livestock production (4, 5).

Furthermore, the selected approach assumes a causal relation between the food risk factors and NCD mortality, despite being largely based on observational nutritional studies with well-characterized drawbacks (6), which could also result in a mis-estimation of the health impact of risk factors. Beyond an effort to use previously published meta-analyses, methods to systematically evaluate the quality of evidence such as GRADE (7) were not used on the evidence base, increasing the possibility of overstating weak or spurious associations. Beyond these fundamental concerns with the authors' approach, we identified multiple methodological issues that we discuss here.

The *Lancet*’s guidelines for authors require a systematic, transparent approach following widely accepted guidelines such as the GATHER statement (8) to ensure objective, replicable results. The methodology presented in Section 1 of the report and appendix, neither systematic nor transparent, does not conform to this peer-review standard. Several studies and meta-analyses are cited in support of both the recommended daily amounts for different foods in the REF diet, and the RRs measuring the association between foods and NCD outcomes that are used to estimate prevented mortality with the REF diet (1, 2). Yet, the method for selecting the cited literature is not provided: inclusion or exclusion criteria are not mentioned, nor is the rationale for choosing between competitive meta-analyses available to parameterize the prevented mortality calculations. For example, the authors did not document why the 2011 meta-analysis by Chan et al. (9) on the association between red meat consumption and risk of colorectal cancer (CRC) was selected over any of the other meta-analyses on the same subject published between 2015 and 2018, e.g. (10–14). Similarly, there appears to be inconsistent use of literature to select and include associations between food groups and health outcomes in the prevented mortality modeling (2). For example, while the protective effect of nuts against type II diabetes mellitus was included, the effect of low dairy consumption on CRC, identified as a risk factor by the Global Burden of Disease project (3), was not applied. Moreover, how the evidence was used to arrive at the dietary composition and daily intake estimates is not described, but our comparisons indicate that some of the recommended daily amounts do not match the cited evidence. For instance, the report recommends daily poultry intakes of 29 g (range 0–58 g/d). Yet, in the studies cited in the report, poultry consumption was not significantly associated with negative health outcomes even at higher consumption amounts (≥80 g/d compared with 5–9.9 g/d) (15), and was indeed found to have significant protective effects against CRC and cardiovascular disease in some studies (e.g., at 17–111 compared with ≤16 g/d).

The National Academies of Sciences recent consensus study (16) states that scientifically valid dietary guidelines must be based on high-quality studies. This requires careful assessment of published literature and improved systematic review and meta-analysis methods. Using different quality assessment methodology could change nutritional recommendations, as exemplified by the recent study by Johnston et al., which used the GRADE system to assess strength of evidence on the health effects of red meat consumption and came to conclusions that conflict with other recent nutritional guidelines (17).

We also noted discrepancies in the RRs used in the analysis by Springmann et al. (2). First, the RR values used for the association between red meat consumption and incidence of both total stroke and type 2 diabetes mellitus do not match those in the cited meta-analyses. The total red meat RR for type 2 diabetes mellitus reported by Feskens et al. (18) was 1.13 (95% CI: 1.03, 1.23) but the RR used by Springmann et al. was 1.15 (1.07, 1.24), and the total red meat RR for stroke reported in Chen et al. (19) was 1.15 (1.05, 1.25), but the reported value from Springmann et al. was 1.1 (1.05, 1.15) (2).

Second, as the REF diet does not contain processed meat, the appropriate RR to use for its exposure level would be for fresh red meat, and not total red meat (which includes both fresh and processed red meats). This would change the RRs used for red meat consumption and type II diabetes mellitus under the REF diet from 1.13 (1.03, 1.23) to 1.15 (0.99, 1.33) (18), a nonsignificant association. To be consistent in approach then, similar to coronary heart disease which has a significant association with processed but not with fresh red meat (20), type II diabetes mellitus should not be included in the health outcomes attributed to red meat consumption (2). The RR applied to red meat for CRC and for stroke would also be changed under the REF diet, as the fresh red meat RRs for these are 1.17 (1.05, 1.31) (9) – rather than 1.14 (1.04, 1.24) for total red meat and CRC – and 1.13 (1.01, 1.25) for stroke and fresh red meat (19).

Another important issue with the report is the treatment of statistical uncertainty in key model parameters. The authors state that the health impact RR was the greatest source of uncertainty and thus they only included this RR uncertainty in their estimation of avoided mortalities (2). By doing so, they omitted assessing the impact of other relevant sources of uncertainty such as disease-specific mortality rates, percentage of population consuming each food, amount of each food consumed, and prevalence of underweight, overweight, and obesity in the population.

Last but not least, the REF diet in the report (1) [as well as the vegan, vegetarian, and pescatarian diets (2)] has an ideal calorie intake that assumes changes in energy consumption and thus eliminates underconsumption or overconsumption with perfect adherence in the population. In contrast, the business-as-usual diet compared against it reflects variable energy intakes among country populations and is higher in many countries. The prevented mortality estimations attributed to the REF diet are thus a combination of the effect of adjusting energy consumption and the impact of dietary composition. Yet, the authors did not statistically assess the relative contribution of weight-related (i.e., energy consumption) compared with food-specific factors (i.e., dietary composition) on the overall prevented mortality.

Considering the issues above, we attempted to replicate the report's findings. Using the available information on methods and data (2), optimization algorithms to back-calculate missing inputs, and results available through the Oxford Data Archive, we approximately reconstructed the estimates of prevented mortality in the United States assuming a population-wide change to the REF diet. We then assessed the impact on the results of correcting two of the main problems discussed above, with use of the appropriate RRs and including all sources of uncertainty in the mortality calculations. To be consistent with Springmann et al.’s procedures, a RR of 1 was applied for type II diabetes mellitus and red meat, removing it from the health impacts. The RR used for stroke under the current diet was 1.15 (1.04, 1.28), and under the REF diet was 1.13 (1.01, 1.25), and the RR for total red meat and for unprocessed red meat and CRC were applied as described above, 1.17 (1.05, 1.31) for the reference diet exposure to unprocessed meat only and 1.14 (1.04, 1.24) for the current diet.

Mortality rate uncertainty was derived from the Global Burden of Disease project (3). Food consumption uncertainties were estimated using current data from the United States as the estimates used by Springmann et al. were not publicly available. These changes increased the uncertainty reported in the overall prevented mortalities, from an SE of 243 to one of 812, changing the distribution of prevented deaths as compared in **Figure 1**.

**FIGURE 1:**
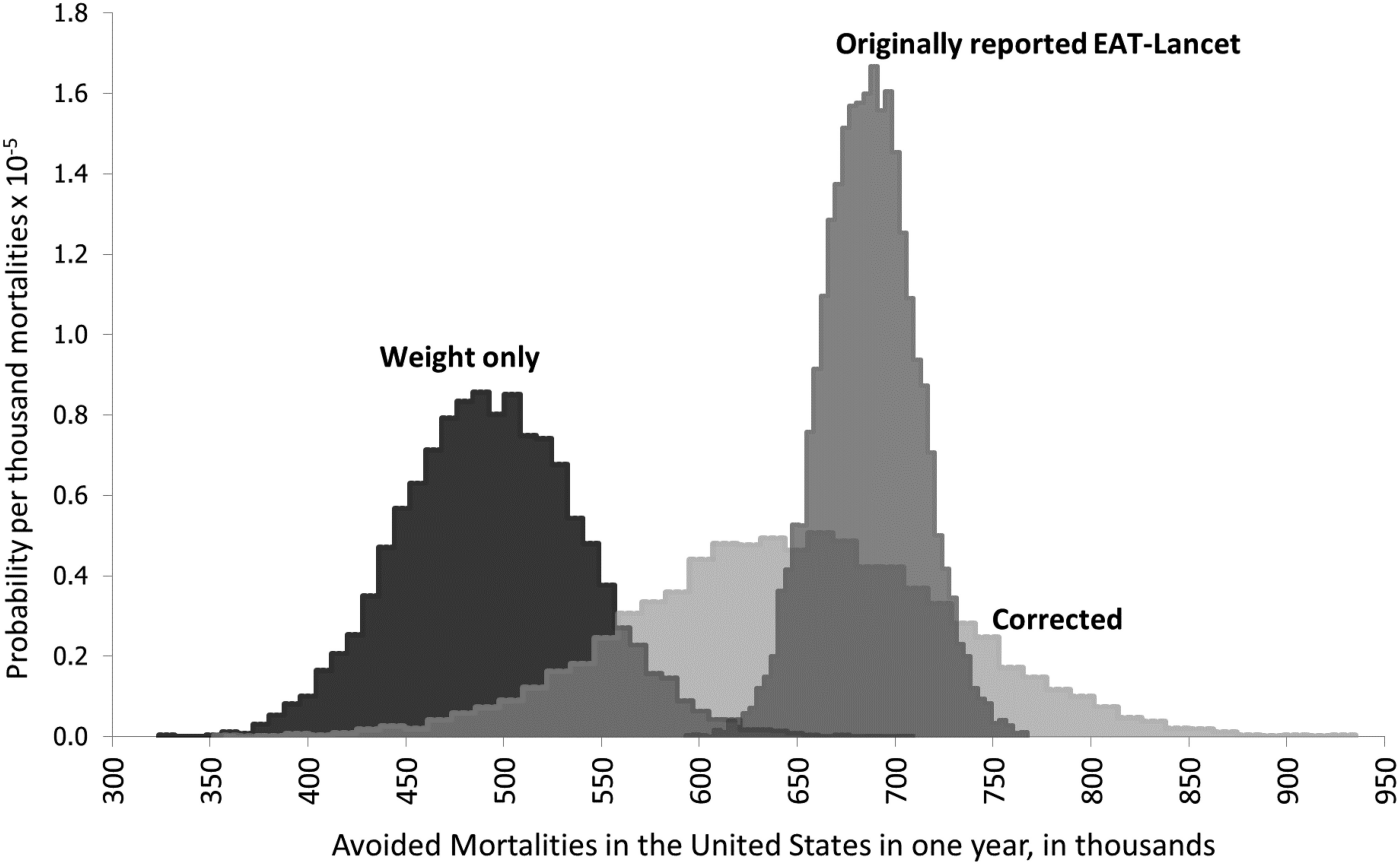
Distributions of avoided mortalities for 2030 in the United States from the reference (or flexitarian) diet compared with the baseline (business as usual) diet (lightest gray), with added uncertainty and corrected risk ratios. Avoided mortality from elimination of underweight, overweight, and obesity (darkest gray) in the reference (or flexitarian) diet was compared with the reported distribution of overall avoided mortalities in the United States (medium gray) from Springmann et al., 2018 (2).

Despite the report describing “high scientific certainty” in the impacts of the REF diet (1), we found that with corrections to both the uncertainty and RRs, but not with changes to either alone, the total avoided mortalities may not be statistically significantly different from the avoided mortalities that are strictly caused by weight-related risk factors. In the EAT-Lancet diet, the calorie intake of all individuals is assumed as fixed such that underweight, overweight, and obese prevalence in the US population is eliminated, and this assumption of change in energy consumption is responsible for 63–94% of the predicted total mortalities avoided in the United States. That is, after adjusting for the omitted uncertainty and RR errors, and beyond the impact of changing energy consumption to a fixed and ideal level with the REF diet, there may not be statistically significant changes in deaths from switching from the current diet composition to the REF diet. Our analysis shows that, when appropriate RRs are used, parameter uncertainty is sufficiently large to change the result interpretation for an individual country and thus, a complete quantification of uncertainty should be incorporated in the global analysis. The discussion of appropriate certainty levels to establish dietary recommendations is ongoing, and with differences in risk management approaches globally, a harmonized solution is elusive. Nonetheless, a transparent discussion of these issues requires appropriately quantified uncertainty (21) and transparent, up-to-date methods for evidence evaluation.

Food production methods and dietary habits impact both human health and the environment in many ways, and optimal solutions to these multifaceted issues should be sought. However, the evaluation of these solutions must be replicable, follow scientific standards, and incorporate statistical uncertainty, or they risk inspiring policies that are costly and possibly ineffective.

## Notes

Supported by a contract from MatPrat Norway. The funders had no role in the design, analysis, or decision to publish this work.

Author disclosures: SC, JGP, and FJZ report, outside of the submitted work, grants from the Beef Checkoff, through National Cattlemen's Beef Association, and from the USDA Foreign Agricultural Service. FJZ is a member of the US National Advisory Committee on Microbial Criteria for Foods. Also, as part of his work with EpiX Analytics, FJZ reports consulting engagements outside the submitted work, including USDA food safety and inspection services, UK Food Standards Agency, Hawaii department of agriculture, and DSM nutritional products. SC reports consulting engagements outside the submitted work, including for Hawaii department of agriculture and Teva pharmaceuticals. JGP reports consulting engagements outside the submitted work, including for Tillotts Pharma.

Abbreviations used: CRC, colorectal cancer; NCD, noncommunicable diseases; REF, reference diet; RR, relative risk; SDI, sociodemographic index
